# SUPPORT Tools for evidence-informed health Policymaking (STP) 14: Organising and using policy dialogues to support evidence-informed policymaking

**DOI:** 10.1186/1478-4505-7-S1-S14

**Published:** 2009-12-16

**Authors:** John N Lavis, Jennifer A Boyko, Andrew D Oxman, Simon Lewin, Atle Fretheim

**Affiliations:** 1 Centre for Health Economics and Policy Analysis, Department of Clinical Epidemiology and Biostatistics, and Department of Political Science, McMaster University, 1200 Main St. West, HSC-2D3, Hamilton, ON, Canada, L8N 3Z5; 2 Health Research Methodology PhD Programme, 1200 Main St. West, HSC-2D1, Hamilton, ON, Canada, L8N 3Z5; 3 Norwegian Knowledge Centre for the Health Services, P.O. Box 7004, St. Olavs plass, N-0130 Oslo, Norway; 4 Norwegian Knowledge Centre for the Health Services, P.O. Box 7004, St. Olavs plass, N-0130 Oslo, Norway; Health Systems Research Unit, Medical Research Council of South Africa; 5 Norwegian Knowledge Centre for the Health Services, P.O. Box 7004, St. Olavs plass, N-0130 Oslo, Norway; Section for International Health, Institute of General Practice and Community Medicine, Faculty of Medicine, University of Oslo, Norway

## Abstract

*This article is part of a series written for people responsible for making decisions about health policies and programmes and for those who support these decision makers*.

Policy dialogues allow research evidence to be considered together with the views, experiences and tacit knowledge of those who will be involved in, or affected by, future decisions about a high-priority issue. Increasing interest in the use of policy dialogues has been fuelled by a number of factors: 1. The recognition of the need for locally contextualised 'decision support' for policymakers and other stakeholders 2. The recognition that research evidence is only one input into the decision-making processes of policymakers and other stakeholders 3. The recognition that many stakeholders can add significant value to these processes, and 4. The recognition that many stakeholders can take action to address high-priority issues, and not just policymakers. In this article, we suggest questions to guide those organising and using policy dialogues to support evidence-informed policymaking. These are: 1. Does the dialogue address a high-priority issue? 2. Does the dialogue provide opportunities to discuss the problem, options to address the problem, and key implementation considerations? 3. Is the dialogue informed by a pre-circulated policy brief and by a discussion about the full range of factors that can influence the policymaking process? 4. Does the dialogue ensure fair representation among those who will be involved in, or affected by, future decisions related to the issue? 5. Does the dialogue engage a facilitator, follow a rule about not attributing comments to individuals, and *not *aim for consensus? 6. Are outputs produced and follow-up activities undertaken to support action?

## About STP

*This article is part of a series written for people responsible for making decisions about health policies and programmes and for those who support these decision makers. The series is intended to help such people ensure that their decisions are well-informed by the best available research evidence. The SUPPORT tools and the ways in which they can be used are described in more detail in the Introduction to this series *[1]. *A glossary for the entire series is attached to each article (see Additional File *1*). Links to Spanish, Portuguese, French and Chinese translations of this series can be found on the SUPPORT website http://www.support-collaboration.org. Feedback about how to improve the tools in this series is welcome and should be sent to: STP@nokc.no*.

## Scenarios

*Scenario 1: You are a senior civil servant and have been invited to a policy dialogue about an issue that is of growing interest to the Minister. You are concerned about whether the policy dialogue is being organised in way that will inform different elements of the issue, and recognises the importance of drawing on both research evidence and stakeholder views and experiences. You also want to ensure that the policy dialogue does not conclude with a recommendation that is politically or economically unfeasible and hence potentially awkward for the Minister concerned*.

*Scenario 2: You work in the Ministry of Health and have been given a few hours to prepare an assessment of a planning document for a policy dialogue that will address a high-priority issue for the Ministry. All that you have been told is that this policy dialogue is different in a number of ways from the type of stakeholder engagement processes that you have organised in the past for the Ministry, including how it will be informed by a pre-circulated summary of the best available research evidence on the problem, options to address it, and implementation considerations*.

*Scenario 3: You work in an independent unit that supports the Ministry of Health in its use of research evidence in policymaking. You are organising a policy dialogue for senior Ministry officials and key stakeholders to deliberate about a problem, options to address it, and implementation considerations. You have been told to organise the policy dialogue in a way that is likely to enhance its potential impact, but you want guidance on how to do so*.

## Background

For policymakers (Scenario 1), this article suggests a number of questions that they might ask their staff to consider when deciding whether to participate in a policy dialogue or how to maximise the value of a policy dialogue that they are sponsoring. For those who support policymakers (Scenarios 2 and 3), this article suggests a number of questions to guide their assessment of a plan for a policy dialogue or their organisation of one.

There has been growing interest in identifying interactive knowledge-sharing mechanisms that allow research evidence to be brought together with the views, experiences and tacit knowledge of those who will be involved in, or affected by, future decisions about high-priority issues [2,3]. This interest has been fuelled by a number of developments:

1. The recognition of the need for locally contextualised 'decision support' for policymakers and other stakeholders [4,5]

2. The recognition that research evidence is only one input into the decision-making processes of policymakers and other stakeholders [6,7]

3. The recognition that many stakeholders can add significant value to these processes [8,9], and

4. The recognition that many stakeholders can take action to address high-priority issues - not just policymakers

Policy dialogues constitute a promising 'interactive knowledge-sharing mechanism'. The development of these dialogues has been informed, at least in part, by findings from two systematic reviews of the factors influencing the use of research evidence in policymaking [10,11]. While the reviews identified that research in this field was not extensive, rigorous or consistent, a few factors did emerge consistently:

• Higher levels of interaction between researchers and policymakers increased the likelihood of research evidence being used (particularly when the interactions were based on informal relationships). Conversely, a lack of interaction decreased the likelihood of research evidence being used

• Timeliness increased the likelihood of research evidence being used in policymaking, while a lack of timeliness decreased this likelihood

• The likelihood of research evidence being used in policymaking increased when available research evidence accorded with the beliefs, values, interests or political goals and strategies of politicians, civil servants and stakeholders (or when particular political stances had not yet been decided). Conversely, a lack of accord decreased the probability of research evidence being used.

Policy dialogues have the potential to improve the use of research by shaping the factors listed above. This potential can be realised through support related directly to:

1. Interactions between researchers and policymakers (and among a wider range of stakeholders who are able to take action)

2. The timely identification and interpretation of the available research evidence (when a policy dialogue is organised urgently to address a high-priority issue), and

3. The 'real time' identification of accord between research evidence and the beliefs, values, interests or political goals and strategies of policymakers and stakeholders.

Table 1 provides a simple framework for distinguishing the differences between 'dialogue' and 'debate'. While *dialogue *is the goal of policy dialogues, *debate *does not typically offer suitable opportunities for the support of constructive interaction and the identification of shared ground. This does not mean that debate does not have a critical and complementary role in policymaking. Indeed, forums are also needed to enable contesting value positions to be articulated. In these, the extent and quality of the research evidence supporting alternative problem definitions, options, and implementation strategies (supported by very different value positions) can be publicly presented and debated.

**Table 1:** Differences between dialogue and debate

Dialogue	Debate
Collaborative	Oppositional
Common ground	Winning
Enlarges perspectives	Affirms perspectives
Searches for agreement	Searches for differences
Causes introspection	Causes critique
Looks for strengths	Looks for weaknesses
Re-evaluates assumptions	Defends assumptions
Listening for meaning	Listening for countering
Remains open-ended	Implies a conclusion

Source: Adapted from the Co-Intelligence Institute and appearing in Jones CM, Mittelmark MB. The IUHPE Blueprint for Directed and Sustained Dialogue for Partnership Initiatives

Models for policy dialogues can be distinguished in three ways both from each other and from other stakeholder engagement processes in terms of their:

• Goals - which can include information sharing, networking, discussion, consensus statement development, and action planning about related goals and/or processes

• Group composition, and

• Group processes - which can includes pre- and post-circulated materials and format (e.g. concurrent deliberations in several groups or sequential deliberations in a single group, and rules)

Considerable attention has been paid to these distinctions (and their implications) in public engagement initiatives [12,13] and in clinical practice guideline development [14-16]. For example, researchers have developed an evidence base to inform choices about the design of guideline development processes. This includes approaches to panel composition, the format of pre-circulated evidence summaries, and consensus rules [17].

Far less attention has been given to the benefits, harms and costs of alternative approaches to policy dialogues that seek to support evidence-informed policymaking, or to support other types of evidence-informed action related to health systems. A systematic review found no rigorous evaluations of the effects of policy dialogues [3]. However, the review did identify a variety of policy dialogue characteristics that appear promising, including consultation with all parties affected by an outcome, the fair representation of scientists and stakeholders, high-quality syntheses of the scientific evidence, and skilful chairing [2,3]. Our own formative evaluation of a policy dialogue that involved policymakers, civil society groups and researchers from 20 low- and middle-income countries found that pre-circulated evidence summaries, skilled facilitation, the application of the Chatham House Rule (prohibiting the attribution of particular comments), and a lack of emphasis on achieving consensus, were among the highly-valued design features [18].

## Questions to consider

The following questions can guide how to organise and use policy dialogues to support evidence-informed policymaking:

1. Does the dialogue address a high-priority issue?

2. Does the dialogue provide opportunities to discuss the problem, options to address the problem, and key implementation considerations?

3. Is the dialogue informed by a pre-circulated policy brief and by a discussion about the full range of factors that can influence the policymaking process?

4. Does the dialogue ensure fair representation among those who will be involved in, or affected by, future decisions related to the issue?

5. Does the dialogue engage a facilitator, follow a rule about not attributing comments to individuals, and *not *aim for consensus?

6. Are outputs produced and follow-up activities undertaken to support action?

### 1. Does the dialogue address a high-priority issue?

Policy dialogues should ideally address an issue considered high priority by some or all stakeholders. If a particular issue has been on the agenda of key stakeholders for some time, then policy dialogues, like policy briefs (discussed further in Article 13 in this series), may act as a way to spur action [19]. The Evidence-Informed Policy Network (EVIPNet) in both Burkina Faso and Cameroon, for example, convened a national policy dialogue to address the long-standing challenge of low coverage rates for artemisinin-based combination therapies (ACT) to treat uncomplicated falciparum malaria. If an issue in a policy brief is relatively new, then the associated policy dialogue may potentially play an agenda-setting role. But irrespective of whether it does so or not, the focus of a policy dialogue would always ideally be an issue deemed to be a priority by at least some key stakeholders.

The process of obtaining consensus on the selection of a priority issue for a policy dialogue, however, may leave organisers hostage to policymakers and stakeholders who support the status quo or are seeking to avoid change. Issues related to obtaining consensus on how a problem can best be clarified or options best framed, may also privilege those seeking to avoid change. Such groups may be also be privileged by the choice of dialogue invitees or facilitator, and decisions related to follow-up activities to support action. (These concerns from the focus of Questions 2 to 6 below).

While our focus in this article is primarily on policy dialogues organised with the active engagement of existing political regimes, other policy dialogue scenarios are possible. These may include dialogues organised by those working with opposition leaders, 'shadow' health ministers, and others who might not share the prevailing orthodoxy about what constitutes a high-priority issue or a feasible set of approaches to addressing it.

Because of the way in which priorities change, the timing of policy dialogues is also often critical. In order to address issues when they are considered a high priority and 'windows of opportunity' for change are evident, it may be necessary to organise policy dialogues rapidly.

### 2. Does the dialogue provide opportunities to discuss the problem, options to address the problem, and key implementation considerations?

Policy dialogues, like policy briefs, focus on:

1. Different features of a problem, including (where possible) how it affects particular groups

2. Options to address the problem, and

3. Key implementation considerations

During policy dialogues, participants may conclude that none of the options are optimal. In these instances, they may advocate 'borrowing' additional features from other options in order to create a new hybrid (or 'bundled') option. Dialogues may also be convened at different stages of the policymaking process, giving greater focus to *problem *definition earlier in the process and to *implementation *later.

Policy *briefs *present the best available synthesised research evidence. But (as described in Article 13 in the series) they do not speak explicitly to potential actions based on that evidence [19]. Policy *dialogues*, in contrast, can do this. The focus, in these instances, could be on working through what actions can be taken *individually *(by a politician, for example) and *collectively *(by a coalition, for instance, of health professional associations). The fact that this may be done 'collectively', however, does not imply that everyone will be included. It may instead mean that only several of the groups whose members are participating in a policy dialogue will move forward collectively. And as we discuss below, consensus on the type of collective action chosen is usually not actively sought.

### 3. Is the dialogue informed by a pre-circulated policy brief and by a discussion about the full range of factors that can influence the policymaking process?

A policy brief, as described in Article 13 in this series, is a highly efficient way of introducing global *and *local research evidence about a problem [19]. It also provides options to address a problem, as well as introducing key implementation considerations to dialogue participants. The goal of a policy dialogue is to support the full discussion of relevant considerations (including research evidence) about a high-priority issue in order to inform policymaking and other types of action. Dialogues provide a vehicle for harnessing many types of information and creating locally contextualised knowledge that can inform policymaking and other types of action.

To ensure that key relevant research evidence is taken into account, it is important to have policy briefs pre-circulated. This is also critical because policy briefs provide common ground from which discussions about the issues can be launched. At the start of each set of deliberations (about a problem, options and implementation considerations, respectively), highlights from the corresponding section of a policy brief may be introduced informally. These final highlights would ideally be informally presented and discussed. The alternative of a more formal method of presentation may give some participants the impression that research evidence constitutes the sole focus of the deliberations, or takes precedence over other considerations. A final round of deliberations focusing on who may be able to support the implementation of possible actions has no corresponding written section in a policy brief.

While research evidence can be codified in the form of a policy brief, it is perhaps the views, experiences and tacit knowledge of those who will be involved in, or affected by, future decisions about a high-priority issue that can best emerge spontaneously in the course of a policy dialogue. Dialogue participants would ideally be invited to introduce their own understanding about factors that need to be considered. These include on-the-ground realities and constraints, the values and beliefs of citizens and communities, the power dynamics among interest groups, institutional constraints, and considerations related to 'external' factors (such as the broader economy or, in the case of low- and middle-income countries, the strategic priorities of donors). These understandings are particularly important given that they shape participant approaches to a problem, the options they may choose to address the problem, the implementation of the options, and future decisions related to who should undertake particular actions.

### 4. Does the dialogue ensure fair representation among those who will be involved in or affected by future decisions related to the issue?

A policy dialogue would ideally bring together the many parties involved in, or affected by, future decisions related to a high-priority issue in order to ensure fair representation. As a first step, this requires the careful mapping of the full range of stakeholders related to the issue at hand. Stakeholder mapping may be achieved by creating an inventory of role categories specific to the issue. Those involved could include:

• Policymakers (including elected officials, political staff or civil servants) in the national government and/or in sub-national governments if independent public policymaking authority related to the issue exists at the sub-national level. These policymakers may be drawn from many different departments, and not just health or finance departments

• Managers in districts/regions, healthcare institutions (e.g. hospitals), and non-governmental organisations, and other relevant types of organisations

• Staff or members of civil society groups, which could include consumer groups, health professional associations, and industry associations, among others, and

• Researchers in national research institutions, universities, and from other jurisdictions.

In some countries, individuals may play several of these roles concurrently (or have played them sequentially).

As a second step, individuals will need to be carefully selected from the role categories above. Two criteria may be useful:

1. The ability of the individuals to articulate the views and experiences of a particular constituency on the issue, while constructively engaging at the same time with participants drawn from other constituencies and learning from them, and

2. The ability of the individuals to champion the actions that will address the issue within their constituencies

Different political systems will have different traditions relating to which individuals - and how many individuals - will be invited to meetings to discuss high-priority issues. It may or may not be possible, or desirable, to adapt a tradition for a specific policy dialogue. But when determining the number of invitees, a key consideration should be the balanced representation of all key constituencies, on one hand, and the opportunity for all individuals to contribute, on the other hand. A total of between fifteen and twenty participants might achieve such a balance for some issues and in some contexts. A group twice this size might be needed for other issues and in other contexts. In some French-speaking African countries, for example, EVIPNet teams have organised policy dialogues that were even larger (this is discussed further in Table 2). In order to allow all individuals to contribute in these instances, the local organisers included frequent concurrent deliberations among subgroups.

**Table 2:** Policy dialogues about improving malaria treatment

Two EVIPNet teams, one in Burkina Faso and one in Cameroon, convened national policy dialogues to support a full discussion of relevant considerations (including research evidence) about how to support the widespread use of artemisinin-based combination therapy to treat uncomplicated falciparum malaria.
The dialogue in Burkina Faso brought together 38 stakeholders in May 2008 to discuss this problem, three options to address it, and key implementation considerations. The insights derived from the policy dialogue directly informed the preparation of the Burkina Faso government's successful application to the Global Fund to Fight AIDS, Tuberculosis and Malaria.
Held in January 2009, the dialogue in Cameroon included almost twice the number of stakeholders involved in Burkina Faso, and worked through the particular features of the same problem of malaria treatment in Cameroon, three options appropriate to the problem, as well as related implementation considerations. This group was divided into four smaller 'working groups' for each set of deliberations. The dialogue received significant media attention and increased the likelihood of meaningful action in the following months.

Invitation letters to policy dialogue can prove critical to engaging key individuals. The title for a policy dialogue would ideally be worded in a way that will engage invited policymakers and stakeholders and may, for example, take the form of a compelling question. The invitation letter would ideally provide a list of those involved in planning the dialogue and a list of funders (of the organisation convening the dialogue and of the dialogue itself), as well as their affiliations.

### 5. Does the dialogue engage a facilitator, follow a rule about not attributing comments to individuals, and not aim for consensus?

A skilled, knowledgeable and neutral facilitator is required to ensure that a policy dialogue is run well. Skill is needed to keep the deliberations focused on the issue at hand, to ensure that all dialogue participants have a voice in the deliberations, and to challenge constructively any possible misinterpretations of the issue under discussion, and evidence of the other factors that may influence decision making. It is particularly important for the facilitator to guard against the possibility that perceptions about the relative status of participants (whether based on position in an organisation, educational background or other factors) or other considerations such as language barriers, do not privilege some participants in the dialogue over others. An intermediate level of knowledge about the issue at hand *and *the local context is required in order to interpret the contributions of the policy brief and to manage the dynamics during the deliberations. Neutrality is also required in order to ensure that all participants perceive the dialogue as a 'safe harbour' for deliberation and not as a vehicle for facilitators to steer deliberations in a direction that they may prefer.

Arranging such a safe space for deliberation requires some commonly agreed rules to reassure individuals that they may speak frankly and without fear of repercussion in the media - or elsewhere - for having done so. Many policy dialogues follow the Chatham House Rule: "Participants are free to use the information received during the meeting, but neither the identity nor the affiliation of the speaker(s), nor that of any other participant, may be revealed" http://www.chathamhouse.org.uk/about/chathamhouserule/. This rule ensures that dialogue participants feel empowered to act on what they have learned while knowing that their contributions will not be used to hurt them in the future.

*Not *aiming for consensus may seem paradoxical at first. But this is an important provision for many policymakers and stakeholders. Policymakers are ultimately responsible for setting policies. Therefore, while actual policy development typically occurs through a complex set of interactions involving government officials and stakeholders, most policymakers would be very hesitant to commit themselves to one approach after only a single dialogue, or without the opportunity to confer with policymakers in other parts of government or with other stakeholders. Similarly, some stakeholders will need to return to their groups or organisations in order to decide what actions the groups or organisations should take. All of this said, although seeking consensus may not be an appropriate goal in most contexts, consensus can and probably should be embraced if it emerges spontaneously.

### 6. Are outputs produced and follow-up activities undertaken to support action?

Action to improve health is the preferred outcome for policy dialogues, and therefore mechanisms are needed to equip both dialogue participants and others with the tools to support such action. As a minimum, both the policy brief and a high-level summary of the policy dialogue (i.e. a summary of key points rather than a detailed report) should be actively disseminated. The dialogue summary would need to remain true to the Chatham House Rule that requires comments not to be attributed to identified individuals or to individuals with identified affiliations. Under this same rule, the dialogue summary may not include a list of dialogue participants.

Additional steps can also be taken to support any required action. For example, the McMaster Health Forum, a university-based convenor of policy dialogues:

1. Offers dialogue participants the opportunity to participate in a brief video interview in which they can describe the insights drawn from the dialogue, or the actions they see as critical to addressing a high-priority issue. At the same time, it is made clear to them that their personal choice to relax the way in which the Chatham House Rule applies to them does not alter the way in which the rule applies to others

2. Offers a personalised briefing about the implications of the dialogue to key stakeholder groups in order to support their understanding of what the policy brief and dialogue summary mean for them, and

3. Offers a year-long evidence service that brings to attention newly published or newly identified systematic reviews. This provides added momentum to proposed actions or the need for changes. The video interviews and evidence service are all posted on the Forum's website to inspire and inform others.

As with all efforts in this nascent domain, such follow-up activities warrant further evaluation.

Over time, consideration can be given to how policy dialogues might 'fit' with the rest of an evidence-informed policymaking process and whether they can become the norm for important issues.

## Conclusion

Policy dialogues represent a new and evolving approach to supporting evidence-informed policymaking. They are one of many forms of political interaction that could usefully be more evidence-informed. The organisation and use of policy dialogues continues to evolve through practical experience. Evaluations of this approach are needed in order to improve our understanding of which particular design features and follow-up activities are well received for particular types of issues and in particular types of contexts. For example, the Chatham House Rule may be perceived as being particularly important for highly politicised topics. Similarly, the objective of *not *aiming for consensus may be perceived as inappropriate in political systems that have a long tradition of civil society engagement in policymaking. Evaluations are also necessary as a way of improving our understanding of whether, and how, policy dialogues and related follow-up activities support evidence-informed policymaking. Table 3 provides a description of one approach to the formative evaluation of policy dialogues.

**Table 3:** An example of an approach to the formative evaluation of policy dialogues

• The McMaster Health Forum surveys participants in all of the policy dialogues it convenes and has the long-term goal of identifying *which *particular design features work best for *which *particular types of issues and in *which *types of health system contexts. Participation is voluntary, confidentiality is assured, and anonymity safe-guarded
• Dialogues are characterised by twelve features and these are the focus of the questions in the formative evaluation survey. A dialogue:
• Addresses a high-priority issue
• Provides an opportunity to discuss different features of the problem, including (where possible) how these affect particular groups
• Provides an opportunity to discuss three options to address the policy issue
• Provides an opportunity to discuss key implementation considerations
• Provides an opportunity to discuss who might take what action
• Is informed by a pre-circulated evidence brief that mobilises both global and local research evidence about the problem, three options to address the problem, and key implementation considerations
• Is informed by a discussion about the full range of factors that can inform how to approach the problem, options to address the problem, and the implementation of these options
• Brings together many parties who would be involved in, or affected by, future decisions related to the issue
• Ensures fair representation among policymakers, stakeholders, and researchers
• Engages a facilitator to assist with the deliberations
• Allows for frank, off-the-record deliberations by following the Chatham House Rule: "Participants are free to use the information received during the meeting, but neither the identity nor the affiliation of the speaker(s), nor that of any other participant, may be revealed", and
• Does *not *aim for consensus
• For each design feature the survey asks:
• How useful did they find this approach on a scale ranging from 1 (Worthless) to 7 (Useful)?
• Comments and suggestions for improvement?
• The survey also asks:
• How well did the policy dialogue achieve its purpose, namely to support a full discussion of relevant considerations (including research evidence) about a high-priority issue in order to inform action on a scale from 1 (Failed) to 7 (Achieved)?
• What features of the dialogue should be retained in future?
• What features of the dialogue should be changed in future?
• What others can do better or differently to address the high-priority issue and what they personally can do better or differently?
• Their role and background (so that the McMaster Health Forum can determine if different groups have different views about, and experiences with, the dialogues)
• The McMaster Health Forum also plans to conduct brief follow-up surveys six months after a dialogue, with the objective of identifying what, if any, actions have been undertaken by dialogue participants and what, if any, impacts have been achieved. Here again, participation is voluntary, confidentiality is assured, and anonymity safeguarded
• The Evidence-Informed Policy Networks (EVIPNet) operating in Africa, Asia and the Americas plan to use a similar approach in the formative evaluation of their policy dialogues

## Resources

### Useful documents and further reading

- Lomas J, Culyer T, McCutcheon C, McAuley L, Law S: *Conceptualizing and Combining Evidence for Health System Guidance*. Ottawa, Canada: Canadian Health Services Research Foundation; 2005 [3]http://www.chsrf.ca/other_documents/pdf/evidence_e.pdf

### Links to websites

- Chatham House: http://www.chathamhouse.org.uk - Source of the 'Chatham House Rule'

- Evidence-Informed Policy Networks: http://www.evipnet.org - Network of groups involved in convening national policy dialogues.

## Competing interests

The authors declare that they have no competing interests.

## Authors' contributions

JNL prepared the first draft of this article. JB, ADO, SL and AF contributed to drafting and revising it.

## Acknowledgements

Please see the Introduction to this series for acknowledgements of funders and contributors. In addition, we would like to acknowledge Donna Friedsam and Gill Walt for helpful comments on an earlier version of this Article.

This article has been published as part of *Health Research Policy and Systems *Volume 7 Supplement 1, 2009: SUPPORT Tools for evidence-informed health Policymaking (STP). The full contents of the supplement are available online at http://www.health-policy-systems.com/content/7/S1.

## Supplementary Material

Additional file 1 Glossary Click here for file
